# The Effect of Peginterferon Alpha-2a vs. Peginterferon Alpha-2b in Treatment of Naive Chronic HCV Genotype-4 Patients: A Single Centre Egyptian Study

**DOI:** 10.5812/hepatmon.10069

**Published:** 2013-05-28

**Authors:** Maissa El Raziky, Waleed Fouad Fathalah, Wafaa Ahmed El-akel, Ahmed Salama, Gamal Esmat, Mahassen Mabrouk, Rabab Mamoun Salama, Hany Mahmoud Khatab

**Affiliations:** 1 Department of Endemic Medicine and Hepatology, Cairo University, Cairo, Egypt; 2 Department of Pathology, Cairo University, Cairo, Egypt

**Keywords:** Chronic Hepatitis C, Peginterferon-Alpha- 2a, Peginterferon-Alpha- 2b

## Abstract

**Background:**

Egypt has one of the highest (16-8%) prevalence rates of HCV infection in the world. Approximately 90% of Egyptian HCV isolates belong to a single subtype (4a), which responds less successfully to interferon therapy than other subtypes. Studies comparing the efficacy and safety of PEGIFN alfa-2a and PEGIFN alfa-2b in treatment-naive HCV-infected patients have shown conflicting results.

**Objectives:**

Assessing the effects of Peginterferon alpha-2a versus Peginterferon alpha-2b on the sustained virological response in naive chronic HCV genotype-4 Egyptian patients.

**Patients and Methods:**

This retrospective study cohort consists of 3718 chronic HCV patients admitted to a large, Egyptian medical center. 1985 patients had been treated with PEG-IFN alfa-2a plus RBV and 1733 patients with PEG-IFN alfa-2b plus RBV between years 2007-2011. Efficacy outcomes were sustained virologic response (SVR) and treatment discontinuation rates due to serious adverse effects.

**Results:**

The ETR & SVR in patients treated with PEGIFN alfa-2a was 64.1% and 59.6% as compared to treatment with PEGIFN alfa-2b where these parameters were 58.2% and 53.9% respectively (P < 0.05). Treatment discontinuation rates, were similar in the two types of PEGIFN [0.66 (0.37-1.16); P = 0.15]. Significant dose reduction was evident with peginterferon alfa-2b (35.3%) than peginterferon alpha-2a (27.3 %) (P < 0.01). Patients with lower base line AFP and ALT were most likely to achieve SVR using INF alpha 2-a.

**Conclusions:**

Peginterferon alpha-2a has a higher efficacy regarding ETR and SVR as compared to Peginterferon alfa-2b in treatment of naive chronic HCV genotype-4 patients.

## 1. Background

Hepatitis C virus (HCV) infection is increasingly becoming a major public health problem, threat and concern worldwide (1). There are 170 million infected individuals worldwide, i.e., the prevalence of infection is nearly 3% (2). Most of them are chronically infected and are at risk of liver cirrhosis and hepatocellular carcinoma (HCC) (3). Egypt has one of the highest (16-18%) prevalence rates of HCV infection in the world (4). Approximately 90% of Egyptian HCV isolates belong to a single subtype (4a), which responds less successfully to interferon therapy than other subtypes (5). Several meta-analyses and systematic reviews confirm that a combination of pegylated IFN with ribavirin is effective in treating patients with CHC, leading to high levels of SVR (6). Two forms of peginterferon, peginterferon alfa-2a (40KD) (Pegasys, Hoffmann-La Roche) and peginterferon alfa-2b (12KD) (PegIntron, Schering-Plough Corporation), are commercially available, which differ in terms of their pharmacokinetic, viral kinetic and tolerability profiles (7). The optimal duration of treatment for patients with genotype 1 or 4 is 48 weeks. For patients with genotype 2 or 3, 24 weeks has been the standard (8). Consensus guidelines have recommended the use of either peginterferon alfa-2b or peginterferon alfa-2a in combination with ribavirin for the treatment of chronic hepatitis C; however this was based on a number of non-comparative studies demonstrating similar safety and efficacy between the two treatments (9). Moreover, comparative studies between the peginterferons with respect to structural modifications and dosing (weight-adjusted vs. fixed) may demonstrate important differences in clinical outcomes, and there has been some evidence of such differences (10).

## 2. Objectives

The aim of this study was to assess the effects of Peginterferon alpha-2a versus Peginterferon alpha-2b on the sustained virological response in naive chronic HCV genotype-4 patients.

## 3. Patients and Methods

### 3.1. Type of the Study

This was a retrospective study conducted between years 2007-2011, at a single center, as a part of a national program, which focused on chronic HCV patients recruited from Cairo-Fatemic Hospital, Egyptian ministry of health and population (MOHP) (located in Cairo, Egypt). Funding of investigations and treatment expenses was provided by the MOHP.

### 3.2. The Study Sample

This study consists of 3718 Egyptian chronic HCV patients from Cairo-Fatemic Hospital; one of the largest centers for treatment of viral hepatitis affiliated to the MOHP. Subjects were adult patients aged 18-60 years old who had serological, virological and histopathological evidence of HCV infection. Inclusion criteria for patients’ enrollment were according to the national guidelines:

• Adult HCV patients of both sexes who were positive for HCV antibodies using a third generation ELISA test and detectable HCV RNA expressed in IU/ml and measured by COBAS® AmpliPrep/COBAS® TaqMan® HCV assay which utilizes real-time reverse transcriptase PCR to measure HCV viral load over a broad dynamic range

• Hematological profile: white blood cell (WBC) > 4.000/mm^3^, neutrophil count > 2.000/ mm^3^, platelets > 100.000/ mm^3^

• Biochemical liver profile: direct bilirubin of 0.3 mg/dl or within 20% of ULN, PT < 2 seconds above ULN, albumin > 3.5gm/dl

• Normal kidney function tests and controlled blood sugar in diabetic patients.

• TSH within normal range and ANA titer < 1:20.

Patients with decompensated liver disease, co-infected patients with HBV (positive HBsAg), co-infected patients with HIV (positive HIV-1 or HIV-2), patients with hepatocellular carcinoma, patients with severe psychiatric disease and patients with serious co-morbid conditions were excluded from the current study. 7920 patients were presented to us and only 3718 patients were enrolled in the study.

### 3.3. Enrollment Assessment

A standardized enrolment questionnaire was completed by patients’ physicians. Each patient was subjected to a detailed medical history and clinical assessment for proper selection and exclusion of those with absolute contraindications. Each treating physician was responsible for collecting the clinical, laboratory data as well as taking baseline blood specimens after obtaining a written informed consent from each patient. The consent was designed to explain the treatment process that might extend up to 48 weeks, importance of adherence to treatment, possible adverse effects and expected response rates.

### 3.4. Laboratory Procedures

Blood tests performed as part of the enrollment process included complete blood picture, quantitative HCV-RNA by PCR, liver enzyme; (AST, ALT), serum albumin and INR, TSH, HBsAg, HIV-1, HIV-2, ANA and anti-schistosomal antibodies. We calculated raised AST and ALT ratios based on denominators that were at the upper end of the quoted normal range. All laboratories performed regular quality controls to assure validity of their results. Liver biopsies were obtained by ultrasonography guidance after checking the adequacy of the patients´ coagulation profile. Metavir scoring method was used for histopathological assessment (11).

### 3.5. Randomization Method

A random allocation for allocating patients in to the two groups was used. Participants were assigned to the treatment groups after reviewing the eligibility criteria. The group used one treatment type was assigned to one week and the next week and the other treatment type was assigned on the regular basis. Participants who fulfilled the eligibility criteria received the treatment type assigned for the corresponding week. This was an open label study. Patients were randomized to receive either 180 µgm/week of peginterferon alpha 2a (Pegasys) subcutaneously plus 15 mg/kg/day of ribavirin orally or 1.5 mcg/kg/week of peginterferon alpha 2b (Peg-intron) subcutaneously plus 15 mg/kg/day of ribavirin orally.

### 3.6. Follow Up

Virological parameters (HCV-RNA): baseline, weeks 12, 24, 48 and 72 were tested. Patients not fulfilling EVR at 12 weeks had discontinued treatment. Clinical and laboratory follow up data, which reported possible adverse side effects and treatment responses were evaluated. A threefold increase in AST or ALT was considered a significant elevation, and prompted a reduction in the dose of interferon by half. Also reduction in absolute neutrophilic count below 1000/mm^3^ prompted reduction in the dose of interferon by half. TSH abnormalities mean an increase in blood level and for those patients endocrinal consultation was done and no reduction in treatment dose or discontinuation was recommended. The duration of follow up for relapse cases and side effects was 72 weeks for compliant patients.

### 3.7. Patients Consent

Informed written consent from each patient and local ethical committee approval were obtained at time of patients’ recruitment. With respect to patients’ confidentiality, patients were represented in the study by numbers. All personal data was concealed. The study protocol conformed to the ethical guidelines of the 1975, Declaration of Helsinki as reflected in a prior approval by the institution’s human research committee.

### 3.8. Data Collection

Ethical approval for data collection was obtained from the Ministry of Health and Population ethics committee. Data were obtained from HCV patients´ medical records since the program inception. Data collection included demographic, laboratory, utlrasonographic data and liver biopsy results. This information was largely taken from medical notes and was checked for completeness and correctness. If inconsistencies were found or corrections were thought to be necessary, then the forms were sent back to the physician for amendment. Finally, data were entered into a computer database by a person with paramedical training. All laboratories performed regular quality controls to assure validity of their results.

### 3.9. Data Filtering and Obtaining High Quality Data

Data filtering was applied which is the act of detecting and correcting (or removing) corrupt or inaccurate records from a record set or database in addition to removing typographical errors or validating and correcting values against a known list of entities. The identified incomplete, incorrect, inaccurate or irrelevant parts of the data maybe had been replaced, modified or deleted. High quality data needs to be characterized by accuracy, integrity, completeness, validity, consistency, uniformity and uniqueness. Data Auditing: The data was audited with the use of statistical methods to detect anomalies and contradictions.

### 3.10. Statistical Methods

The descriptive statistics were provided with mean ± SD or median for non parametric data. The χ^2^ test and t-student test were employed for analysis of qualitative or quantitative variables, respectively. Pearson correlation was done to correlate continuous variables, while spearman correlation was performed for correlating fibrosis stages with other variables. In all tests, P values were significant if less than 0.05. Multivariate analysis was used to reveal the independent variables of response, which are beyond the scope of this paper. SPSS (12.0) for Windows was used.

## 4. Results

The two arms of the study were comparable. Demographic features of the studied patients are shown in Table 1 with no significant differences between both treatment groups.

**Table 1.** Demographic Features of the Studied Patients

Variable	Standard Dose Peginterferon α-2a + Ribavirin (n = 1985)		Standard Dose Peginterferon α-2b + Ribavirin (n = 1733)		P value
	No. (%)	Mean ± SD	No. (%)	Mean± SD	
**Gender**					0.32
Male	1593 (80.3)		1413 (81.5)		
Female	392 (19.7)		320 (18.5)		
**Age**		42.10 ±9.816		41.67 ±9.539	0.201
< 50	1430 (78.4)		1268 (79.8)		
> 50	395 (21.6)		321 (20.2)		
**BMI**		28.2487 ±4.27326		28.1758 ±4.33641	0.622
< 24.9	436 (24.2)		375 (24.0)		
25-29.9	743 (41.2)		653 (41.7)		
30-35	518 (28.7)		453 (28.9)		
> 35	105 (5.8)		84 (5.4)		

The laboratory data of the studied patients showed no significant statistical difference between both treatment groups except for AFP and blood glucose levels. (Table 2) Univariate logistic regression analysis (reported as odds ratios [ORs] with 95% confidence intervals [CIs]) showed that serum alpha fetoprotein was an independent variable associated with failure of SVR [OR = 1.1, CI (1.0 - 1.1)] with P < 0.01. HCV RNA, AST, AFP were presented by median and interquartile ranges and compared by Mann-Whitney U test.

**Table 2.** Laboratory Data of the Studied Patients

	Standard Dose Peginterferon α-2a+Ribavirin (n=1985)	Standard Dose Peginterferon α-2b+Ribavirin (n= 1733)	P value
**Glucose, Mean ± SD.**	100.40 ± 28.95	97.67 ± 27.57	0.004
**Creatinine, Mean ± SD.**	0.90 ± 0.20	0.90 ± 0.20	0.896
**Albumin, Mean ± SD.**	4.20 ± 0.47	4.20 ± 0.47	0.808
**Total Bil., Mean ± SD.**	0.80 ± 0.28	0.80 ± 0.29	0.612
**Indirect Bil., Mean ± SD.**	0.58 ± 0.28	0.58 ± 0.27	0.991
**WBC, Mean ± SD.**	6.47 ± 1.83	6.44 ± 1.79	0.642
**ANC, Mean ± SD.**	3.37 ± 1.23	3.39 ± 1.21	0.776
**HB, Mean ± SD.**	14.08 ± 1.53	14.15 ± 1.50	0.213
**PLT, Mean ± SD.**	212.92 ± 63.11	213.87 ± 61.63	0.649
**Prothrombin, Mean ± SD.**	86.71 ± 10.63	86.44 ± 10.67	0.456
**TSH, Mean ± SD.**	1.59 ± 1.00	1.55 ± 0.97	0.254
**ALT (40) , Mean ± SD.**	63.40 ± 43.12	62.91 ± 42.55	0.725
**HCV RNA IU/ML^a^**			0.87
Mean ± SD.	1096757.06 ± 7597981.01	1084419.77 ± 6038879.08	
Median	0.9 x10^5^	0.9 x10^5^	
IQR	4.2 x10^5^	4.0 x10^5^	
**AST (40)^a^**			0.20
Mean ± SD.	58.32 ± 49.16	55.36 ± 34.65	
Median	46.4	46	
IQR	34	33.4	
**AFP (10)^a^**			0.02
Mean ± SD.	6.53 ± 12.27	37.95 ± 1298.38	
Median	3.4	3.24	
IQR	5.1	4.5	
**ALP (290)^a^**			0.57
Mean ± SD.	179.58 ± 168.23	177.57 ± 167.82	
Median	164	164	
IQR	124	116	
**ANA, No. (%)**			0.325
+ve	28 (1.4)	31 (1.8)	
-ve	1929 (97.2)	1685 (97.2)	

^a^Data presented by median Inter Quartile Range (IQR), P values for Whitney U test.

The histopathological features showed a significant statistical difference (P-value < 0.05) between both groups regarding histopathological activity (more prevalent in those treated with Peginterferon α-2a) but this was not the case for the fibrosis scores (Table 3).

**Table 3.** Histopathological Features of the Studied Patients

	Standard Dose Peginterferon α-2a + Ribavirin (n=1985)	Standard dose Peginterferon α-2b + Ribavirin (n= 1733)	P value
	No. (%)	No. (%)	
**Activity**			0.03
A0	1 (0.1)	2 (0.2)	
A1	812 (59.2)	662 (53.6)	
A2	441 (32.1)	456 (36.9)	
A3	118 (8.6)	116 (9.4)	
**Fibrosis Score**			0.97
F0	10 (0.7)	8 (0.6)	
F1	823 (60.2)	739 (59.9)	
F2	275 (20.1)	260 (21.1)	
F3	174 (12.7)	154 (12.5)	
F4	86 (6.3)	73 (5.9)	

**Figure 1.**
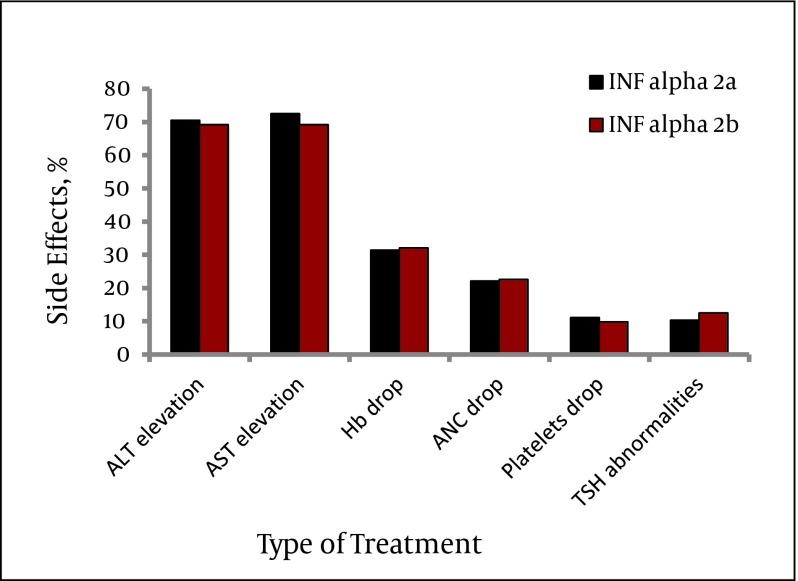
Side Effects in Relation to the Type of Treatment

Most side effects were encountered in the first 12 weeks of initiation of therapy and were related to the interferon therapy; however hemoglobin drop was an effect of interferon and ribavirin. The side effects encountered during treatment were ALT and AST elevation (> 3 fold), hemoglobin, platelets and absolute neutrophilic count drop and TSH abnormalities (increase). Of these side effects, AST elevation and TSH abnormalities were significantly encountered within those treated with Peginterferon α-2b (P value < 0.05). (Figure 1, Table 4)

**Table 4.** Side Effects in Relation to Type of Treatment

Variable	Standard Dose Peginterferon α-2a + ribavirin (n=1985), No. (%)	Standard Dose Peginterferon α-2b + ribavirin (n= 1733), No. (%)	P value
**ALT Elevation**	1400 (70.5)	1199 (69.2)	0.39
**AST Elevation**	1439 (72.5)	1200 (69.2)	0.03
**Hemoglobin Drop**	624 (31.4)	557 (32.1)	0.64
**ANC Drop**	438 (22.1)	391 (22.6)	0.71
**Platelets Drop**	220 (11.1)	216 (9.8)	0.21
**TSH Abnormalities**	205 (10.3)	170 (12.5)	0.04

**Figure 2.**
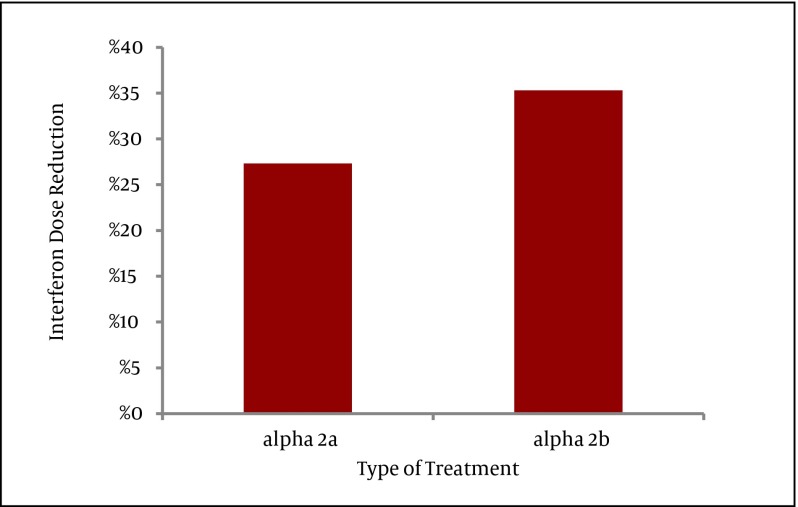
Interferon Dose Reduction in Relation with the Type of Treatment, P value < 0.01

The need for interferon dose reduction was significantly encountered within the group treated with Peginterferon α-2b (P < 0.01) (Figure 2).

**Figure 3.**
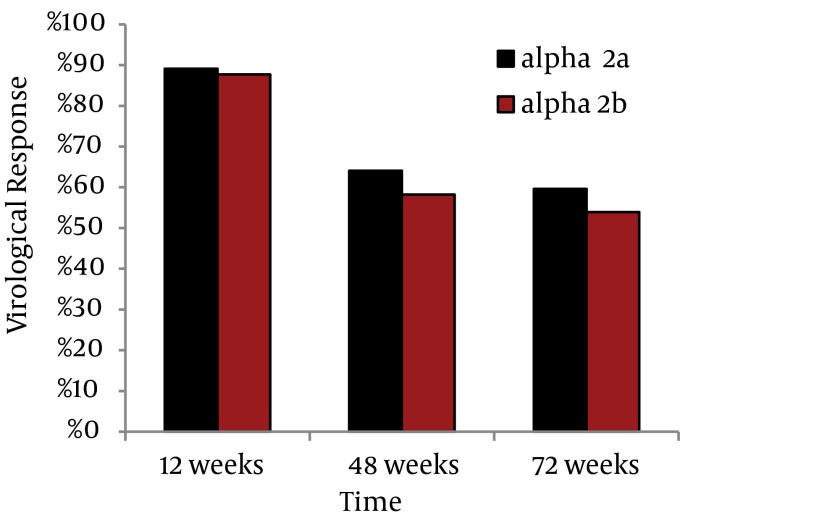
Virological Response in Relation to the Type of Treatment

Regarding the response to treatment in both groups; the early virological response (EVR) was not statistically significant for the two groups (1770 out of 1985 showed EVR for Peginterferon α-2a) (1520 out of 1733 showed EVR for Peginterferon α-2b), however regarding the final treatment response, a statistically significant better response was observed for those treated with Peginterferon alfa-2a (1273 out of 1985 showed ETR for Peginterferon alfa-2a) (1008 out of 1733 showed ETR for Peginterferon alfa-2b) (P < 0.05) (Figure 3). After 48 weeks many patients did not come for follow ups, as there was no further treatment for non-responders. Out of 1273 peginterferon alfa-2a treated patients and 1008 peginterferon alfa-2b treated patients who achieved ETR at week 48, we were able to recruit only 791 and 617 patients respectively at week 72. So we performed a statistical correction to calculate the sustained virological response (SVR). The SVR was significantly higher in the group treated with Peginterferon alfa-2a (P < 0.05) (Figure 3). The relapse rate was 27% for peginterferon alfa-2b and 32% for peginterferon alfa-2a with no significant difference (P > 0.05).

## 5. Discussion

Treatment with peginterferon alfa-2a or peginterferon alfa-2b, plus ribavirin, for 48 weeks is recommended for patients infected with HCV genotype 4, the most common variant in Egypt (5). Despite this recommendation, few data comparing these treatment regimens are available. In our study both treatment groups were comparable in their baseline data, laboratory data and histopathological pattern. Serum AFP was significantly higher in the group treated with peginterferon alpha-2a, and this was a dependent factor associated with failure of response at week 72. Similarly the histological activity was significantly higher in the group treated with peginterferon alpha-2a, however, this was not a dependent factor associated with failure of response at week 72. During the follow up AST elevation and TSH abnormalities were significantly encountered for those treated with peginterferon alpha-2b. Also interferon dose reduction was significantly necessary for those treated with peginterferon alpha-2b. On the contrary some studies found that adverse-event profiles were similar among patients treated with peginterferon alfa-2b or peginterferon alfa-2a (12-14). Another study showed a higher prevalence of neutropenia among patients treated with PEG-IFN alfa-2a (15). In terms of virological response our results declared a significant ETR and SVR in the group treated with peginterferon alfa-2a than the group treated with peginterferon alpha 2-b. This is supported by two meta-analyses which suggested that peginterferon alpha-2a is significantly superior to peginterferon alfa-2b (14, 15) .The first meta-analysis included 7 RCTs and the authors, showed that in the subset of naive patients with genotype 1, 4 and 2, EVR, ETR and SVR were greater for patients treated with PEG-IFN-α2a. Odds Ratios (ORs) were 1.38 (95% confidence interval [CI] 1.11-1.71), 1.67 (95% CI 1.24-2.24), and 1.38 (95% CI 1.02-1.88) respectively. The second meta-analysis included 11 RCTs and the authors, showed that in patients with genotype 1, 4 and 2, overall, peginterferon alpha-2a significantly increased the number of patients who achieved SVR versus peginterferon alfa-2b (47% versus 41%; risk ratio 1.11, 95% confidence interval 1.04-1.19; P =0.004). This was also supported by some RCTs studying HCV genotype-4 patients showing a significant response in those treated with PEG-IFN alfa-2a (16-18). PEG-IFN alfa-2a is considered more cost-effective than PEG-IFN alfa-2b in treatment of chronic HCV patients (19). However other studies showed that the rates of sustained virologic response is similar among patients infected with HCV who received standard-dose or low dose peginterferon alfa-2b or peginterferon alfa-2a, in combination with ribavirin (13, 15). The need for interferon dose reduction was significantly encountered for the group treated with Peginterferon alfa-2b and this might be a factor related to a lower response in this group of patients. It has been shown that high dose interferon induction treatment blocks viral production in over 95% of cases and the effect of interferon dose reduction on viral dynamics can be completely attributed to decrease in the effectiveness of interferon in blocking virion production (20). In our study no significance in relapse rate was found between both treatment groups. However, McHutchison et al. 2009 found that the relapse rate was substantially lower for PEG-IFN alfa-2b than for alfa-2a and that a lower dosage of PEG-IFN alfa-2b was associated with a lower relapse rate (14). Our study has some limitations; first it was only an epidemiological based assumption that all patients are genotype 4 as approximately 90% of Egyptian HCV isolates belong to a single subtype (4a) (5). Second the lack of blinding and the retrospective nature of the study was another limiting factor. Lastly significant number of missing data in SVR cases since some patients didn’t come to follow-ups is of concern, however the latter was statistically correct to reach a definite result. Peginterferon alpha-2a has higher efficacy regarding ETR and SVR as compared to Peginterferon alfa-2b in treatment of naive HCV genotype-4 patients. Significant dose reduction was evident with peginterferon alfa-2b than peginterferon alpha-2a. AST elevation and TSH abnormalities were significantly encountered in the follow up with peginterferon alfa-2b than peginterferon alpha-2a.

## References

[1] Viral hepatitis : Report by the Secretariat. 2009; Available from: http://apps.who.int/gb/ebwha/pdf_files/EB126/B126_15-en.pdf

[2] Schaefer M, Heinz A, Backmund M (2004). Treatment of chronic hepatitis C in patients with drug dependence: time to change the rules?. Addiction..

[3] (1999). Global surveillance and control of hepatitis C. Report of a WHO Consultation organized in collaboration with the Viral Hepatitis Prevention Board, Antwerp, Belgium.. J Viral Hepat..

[4] Darwish MA, Faris R, Darwish N, Shouman A, Gadallah M, El-Sharkawy MS (2001). Hepatitis c and cirrhotic liver disease in the Nile delta of Egypt: a community-based study.. Am J Trop Med Hyg..

[5] Ray SC, Arthur RR, Carella A, Bukh J, Thomas DL (2000). Genetic epidemiology of hepatitis C virus throughout egypt.. J Infect Dis..

[6] Shepherd J, Brodin H, Cave C, Waugh N, Price A, Gabbay J (2004). Pegylated interferon alpha-2a and -2b in combination with ribavirin in the treatment of chronic hepatitis C: a systematic review and economic evaluation.. Health Technol Assess..

[7] Bruno R, Sacchi P, Ciappina V, Zochetti C, Patruno S, Maiocchi L (2004). Viral dynamics and pharmacokinetics of peginterferon alpha-2a and peginterferon alpha-2b in naive patients with chronic hepatitis c: a randomized, controlled study.. Antivir Ther..

[8] Strader DB, Wright T, Thomas DL, Seeff LB (2004). Diagnosis, management, and treatment of hepatitis C.. Hepatology..

[9] Fried MW, Shiffman ML, Reddy KR, Smith C, Marinos G, Goncales FL, Jr. (2002). Peginterferon alfa-2a plus ribavirin for chronic hepatitis C virus infection.. N Engl J Med..

[10] McHutchison J, Sulkowski M (2008). Scientific rationale and study design of the individualized dosing efficacy vs flat dosing to assess optimal pegylated interferon therapy (IDEAL) trial: determining optimal dosing in patients with genotype 1 chronic hepatitis C.. J Viral Hepat..

[11] Bedossa P, Poynard T (1996). An algorithm for the grading of activity in chronic hepatitis C. The METAVIR Cooperative Study Group.. Hepatology..

[12] McHutchison JG, Lawitz EJ, Shiffman ML, Muir AJ, Galler GW, McCone J (2009). Peginterferon alfa-2b or alfa-2a with ribavirin for treatment of hepatitis C infection.. N Engl J Med..

[13] Laguno M, Cifuentes C, Murillas J, Veloso S, Larrousse M, Payeras A (2009). Randomized trial comparing pegylated interferon alpha-2b versus pegylated interferon alpha-2a, both plus ribavirin, to treat chronic hepatitis C in human immunodeficiency virus patients.. Hepatology..

[14] Alavian SM, Behnava B, Tabatabaei SV (2010). The comparative efficacy and safety of peginterferon alpha-2a vs. 2b for the treatment of chronic HCV infection: a meta-analysis.. Hepat Mon..

[15] Awad T, Thorlund K, Hauser G, Stimac D, Mabrouk M, Gluud C (2010). Peginterferon alpha-2a is associated with higher sustained virological response than peginterferon alfa-2b in chronic hepatitis C: systematic review of randomized trials.. Hepatology..

[16] Ascione A, De Luca M, Tartaglione MT, Lampasi F, Di Costanzo GG, Lanza AG (2010). Peginterferon alfa-2a plus ribavirin is more effective than peginterferon alfa-2b plus ribavirin for treating chronic hepatitis C virus infection.. Gastroenterology..

[17] Witthoeft T, Hueppe D, John C, Goelz J, Heyne R, Moeller B (2010). Efficacy and tolerability of peginterferon alfa-2a or alfa-2b plus ribavirin in the daily routine treatment of patients with chronic hepatitis C in Germany: the PRACTICE study.. J Viral Hepat..

[18] Rumi MG, Aghemo A, Prati GM, D'Ambrosio R, Donato MF, Soffredini R (2010). Randomized study of peginterferon-alpha2a plus ribavirin vs peginterferon-alpha2b plus ribavirin in chronic hepatitis C.. Gastroenterology..

[19] Sullivan SD, Craxi A, Alberti A, Giuliani G, De Carli C, Wintfeld N (2004). Cost effectiveness of peginterferon alpha-2a plus ribavirin versus interferon alpha-2b plus ribavirin as initial therapy for treatment-naive chronic hepatitis C.. Pharmacoeconomics..

[20] Bekkering FC, Neumann AU, Brouwer JT, Levi-Drummer RS, Schalm SW (2001). Changes in anti-viral effectiveness of interferon after dose reduction in chronic hepatitis C patients: a case control study.. BMC Gastroenterol..

